# Rosmarinic acid inhibits DNA glycation and modulates the expression of *Akt1* and *Akt3* partially in the hippocampus of diabetic rats

**DOI:** 10.1038/s41598-021-99286-w

**Published:** 2021-10-18

**Authors:** Ameer Alrubaye, Majid Motovali-Bashi, Mehran Miroliaei

**Affiliations:** Department of Cell and Molecular Biology and Microbiology, Faculty of Biological Science and Technology, University of Isfahan, Isfahan, Iran

**Keywords:** Biochemistry, Drug discovery, Genetics, Molecular biology, Diseases, Health care

## Abstract

Non-enzymatic glycation of DNA and the associated effects are among pathogenic factors in diabetes mellitus. Natural polyphenols have anti-diabetic activity. Herein, the protective role of one of the phytochemicals, rosmarinic acid (RA), was evaluated in glycation (with fructose) of human DNA and expression of *Akt* genes in the hippocampus of diabetic rats. In-vitro studies using fluorescence, agarose gel electrophoresis, fluorescence microscopy, and thermal denaturation analyses revealed that glycation causes DNA damage and that RA inhibits it. In-vivo studies were performed by induction of diabetes in rats using streptozotocin. The diabetic rats were given RA daily through gavage feeding. The expression of *Akt* genes (inhibitors of apoptosis) in the hippocampus was evaluated using RT-qPCR. In diabetic rats, *Akt1* and *Akt3* were significantly down-regulated compared to the control group. Treating the diabetic rats with RA returned the expression of *Akt1* and *Akt3* relatively to the normal condition. Past studies have shown that diabetes induces apoptosis in the hippocampal neurons. Given that glycation changes the genes expression and causes cell death, apoptosis of the hippocampal neurons can be due to the glycation of DNA. The results also suggest that RA has reliable potency against the gross modification of DNA under hyperglycemic conditions.

## Introduction

Spontaneous glycation is a non-enzymatic process that occurs naturally under physiological conditions, but it is greatly intensified under hyperglycemic conditions and can be fatal. Glycation causes significant structural and conformational changes in biomolecules, which complicates degenerative diseases, including diabetes^1^. Non-enzymatic glycation proceeds in a step-wise manner and involves Schiff’s base formation and Amadori rearrangements, resulting in the formation of Advanced Glycation End-products (AGEs)^2^. Genetic materials, including DNA, are the biomolecules involved in such a harmful process^3^. Amino groups in nucleic acids can serve as a substrate for the non-enzymatic addition of reducing sugars, leading to some gross alteration in the DNA structure. The source of such non-enzymatic reactions is endogenous sugars, which can induce extensive damage to DNA, leading to much of the cellular mutations^4^. The force imposed by these processes might direct be related to the striking degree of deletions, duplications, insertions, or inter-strand cross-linking^2^.

DNA glycation and its consequences are more severe in diseases concerned with glycation factors aggregation, such as diabetes^5^. In diabetic conditions, fructose concentration increases by the polyol pathway in the independent tissues of insulin such as neural tissue^6–8^. Several studies have shown that experimental diabetes induces apoptosis in the hippocampal neurons^9–12^. The hippocampus is one of the sensitive regions of the brain against metabolic disorders such as diabetes^13^. The serine/threonine kinase Akt, known as protein kinase B (PKB), is a vital part of signal transduction in all eukaryotic cells and as one of the most important protein kinases, it plays a role in inducing cell division and preventing apoptosis^14^. There are three AKT isoforms conserved in the mammalian genome: AKT1 (PKBα), AKT2 (PKBβ), and AKT3 (PKBγ).

Given DNA glycation with different reducing sugars and the possible association of this event with diabetes, the search for tarping agents with the capability of targeting any step of the glycation pathway has always been a health concern. Inhibition of glycation, both in vivo and in food, is vital for promoting human health, especially for the control of diabetes^15^. To this end, bioactive phenols hold a remarkable potential to overcome the toxic effects of sugars^16^. Glycation-induced oxidative stress serves as the key pathogenetic stimulus for oxidative DNA damage, giving rise to characteristic nucleotide adducts^17,18^. Naturally occurring phytochemicals seem to be a helpful strategy to fight such a pernicious process. To find the extent to which the glycation process of DNA can be relieved, the impact of naturally occurring polyphenol, Rosmarinic acid (RA) with documented antioxidant, anti-inflammatory, antibacterial, and anti-apoptotic activities^19,20^, was explored in the fructose-DNA model. This study aimed to evaluate the extent of fructose-mediated AGEs damage to DNA and its inhibition by the interference of RA, as well as evaluation of *Akt* genes expression in the hippocampus of diabetic rats treated with RA. Finally, we tried to explore the probable relationship between apoptosis of hippocampal neurons in diabetic conditions and DNA glycation.

## Results

### Glycation and UV–vis spectroscopic characterization of DNA

**Figure 1:**
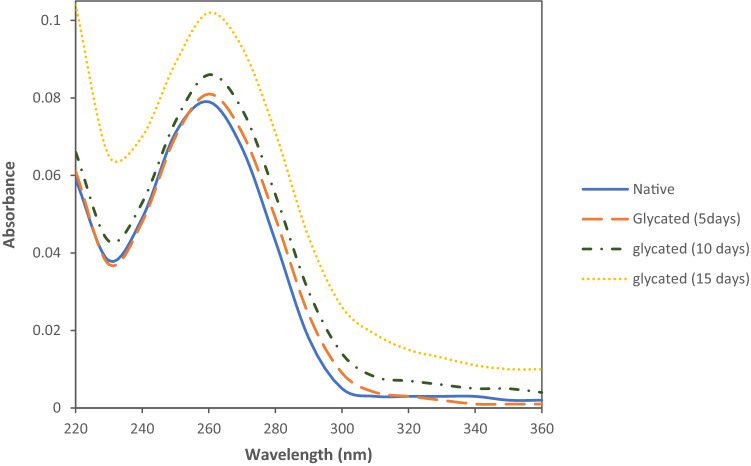
UV absorption of the Native DNA (–) and DNA incubated with fructose 25 mM, after 5 (---), 10 (-.-.) and 15 days (….).

The absorption spectroscopic analysis of the fructose-modified DNA showed a broad absorption band centered at 260 nm with marked hyperchromicity after 15 days of incubation; this was comparable to the native form (Fig. 1). Upon glycation, the trend of kinetic changes at 260 nm kept on rising within 5, 10, and 15 days of incubation in the PBS (pH 7.4). Compared to the 260 nm peak of the native DNA, the hyperchromicities shown by 15 days was 29.11%. Furthermore, the ratio of the intensities at 260:280 nm (A260/A280) was decreased for DNA in the presence of fructose (Table 1).

**Table 1:** Characterization of the native DNA glycated and treated with RA under identical conditions.

Parameter	Native DNA	DNA + Fructose	DNA + Fructose + RA
Absorbance ratio (A260/A280)	1.84	1.44	**–**
Hyperchromicity (%)	**–**	29.11	**–**
Fluorescence intensity	25	110	67
Melting temperature (Tm), °C	82	75	79.3
Zeta potential (Mean) (mV)	− 3.5	− 0.5	− 1.8

### Agarose gel electrophoresis

**Figure 2:**
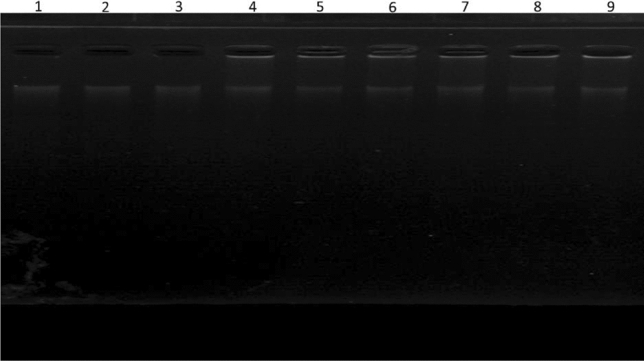
Agarose gel electrophoresis of the glycated DNA incubated with fructose for 5, 10, and 15 days (lane 1–3), the native DNA (lane 4–6), and DNA + Fructose after treating with RA for 5, 10, and 15 days (lane 7–9). Electrophoresis was carried out on the 1.5% agarose gel for 1.5 h at 100 v.

Agarose gel electrophoresis was used to determine the fate of DNA during fructation. Figure 2 demonstrates the electrophoretic analysis of the DNA incubated with fructose when RA was present. The difference in the band pattern and intensity was exceptionally informative. Electrophoretic migration of the native DNA reflected the pattern of the typical genomic DNA which was comparable to the migration pattern of the ligand-treated and modified DNA (Fig. 2). DNA sample not exposed to fructose migrated as a sharp single band initially with the appearance of an additional minor band, whereas the disappearance of the initial band was evident for the fructose-modified DNA (lane 1–3) even after 5 days of exposure. No remarkable changes were observed at the band intensities of the treated samples with RA (lane 7–9); meanwhile, for the samples exposed to fructose at a longer duration (10- and 15-days), their protective effect was substantial against lesions in DNA. The obtained results were comparable for the control samples (lane 4–6) with glycated (lane 1–3).

### Fluorescence studies

**Figure 3:**
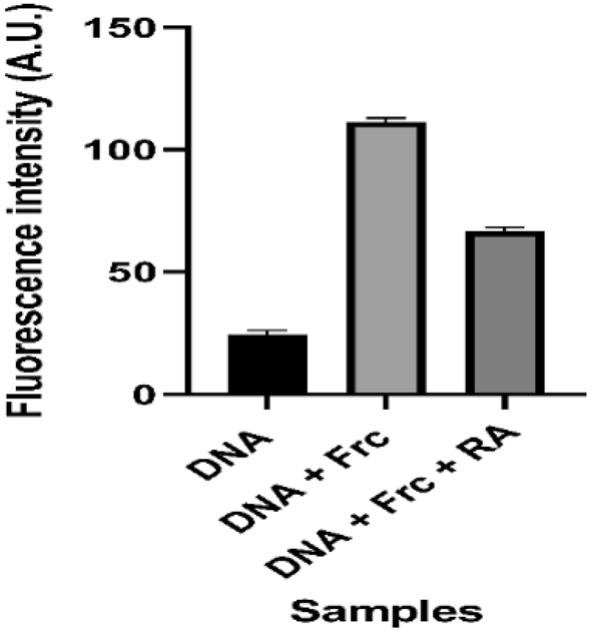
Fluorescence intensity of the native DNA, glycated (DNA + Fructose (Frc)) and treated with rosmarinic acid (RA). The experiments were performed in triplicates.

Glycation causes AGEs to form in DNA along with additional fluorescent adducts that establish disrupt human genes^21^. DNA-AGEs were measured by fluorescence intensity at the excitation/emission wavelength of 370/440 nm. The results obtained by fluorogenic AGEs experiments showed a great extent of alteration in the DNA structure after adding fructose (Fig. 3 and Table 1). It is worth noting that an interesting phenomenon occurred; about 43% of fluorescence intensity was diminished in the treatment with RA (25 µM) (Fig. 3).

### Fluorescence microscopy

**Figure 4:**
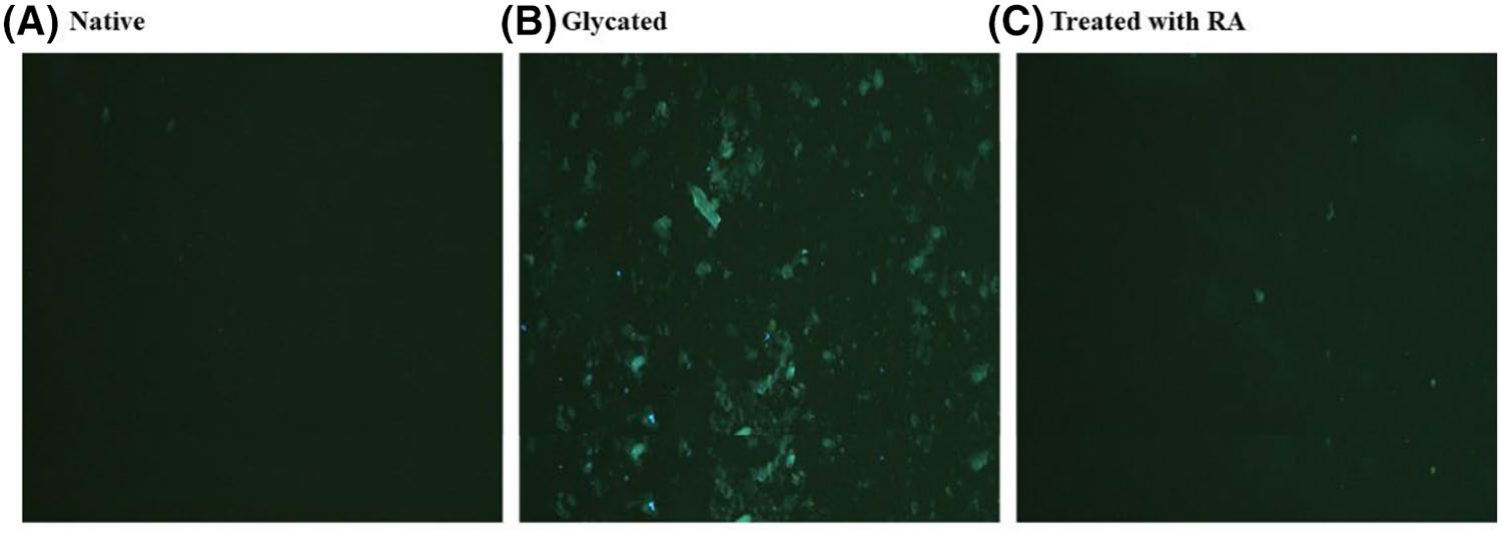
Fluorescence microscopic imaging, the native DNA (**A**), the glycated DNA (**B**), and the glycated DNA in the presence of 25 μM RA (**C**).

To further explore the binding nature of fructose with DNA and evaluate the RA influence, a visual inspection experiment was applied via fluorescence microscopy. As could be seen from the images of Fig. 4, after 15 days of incubation, amorphous-like aggregates were detected in the samples containing DNA-fructose (Fig. 4B), which was comparable to the control (Fig. 4A). Interestingly, after 15 days of incubation, these insoluble aggregates were markedly diminished by the presence of RA (Fig. 4C).

### Zeta potential measurements

**Figure 5:**
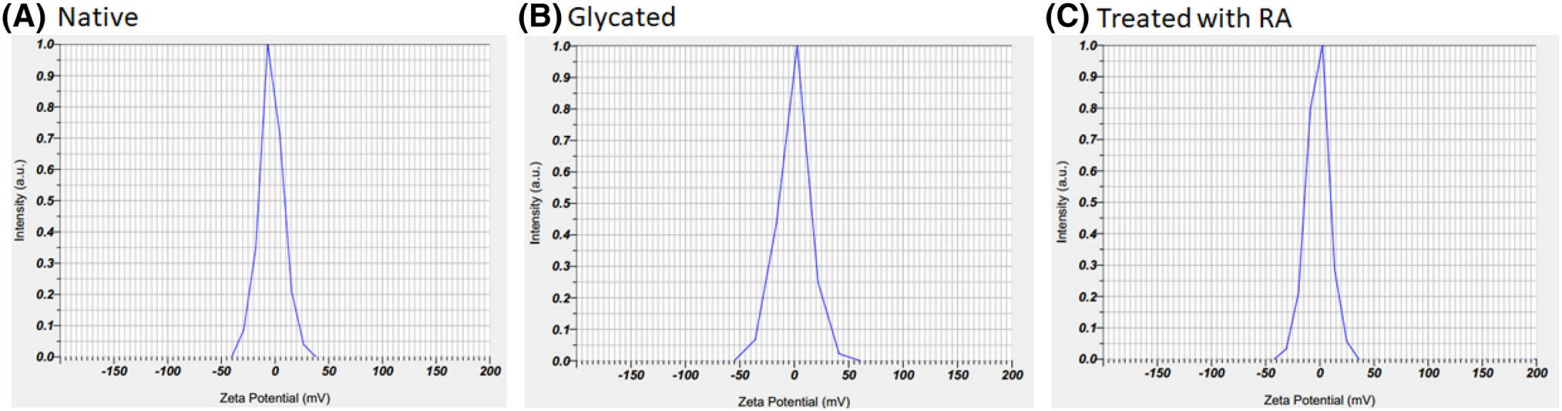
Zeta potential intensity distributions for the native DNA: zeta potential (mean) =  − 3.5 mV (**A**), the glycated DNA: − 0.5 mV (**B**), and the glycated DNA with RA: − 1.8 (**C**).

The zeta-potentials of DNA, Fructose-DNA, and the sample treated with RA were − 3.5 mV, − 0.5 mV, and − 1.8 mV, respectively (Fig. 5 and Table 1). The results of the particle size distribution of RA complexes were still in the nano-size region.

### Thermal denaturation

Thermal denaturation analysis was performed to address the impact of glycation. Based on thermal denaturation analysis, the melting temperature (Tm) for the native, glycated, and RA treated DNA was 82 °C, 75 °C, and 79.3 °C, respectively (Table 1). Therefore, a decrease was observed in the Tm of the modified DNA.

### Quality of the isolated RNA

**Figure 6:**
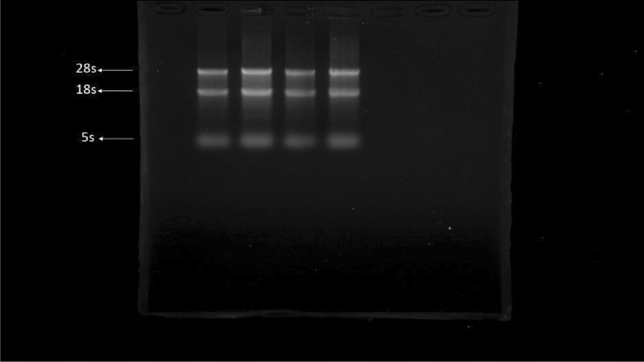
RNA samples isolated from hippocampus electrophoresed on a 2% agarose gel. The presence of three bands 5 s, 18 s, and 28 s are detectable.

After extraction of total RNA from hippocampal tissue, samples were loaded on 2% agarose gel. The presence of three high-resolution 28 s, 18 s, and 5 s bands indicates the quality of the isolated RNA (Fig. 6).

### Downregulation of *Akt1* and *Akt3* in the diabetic rat hippocampus

**Figure 7:**
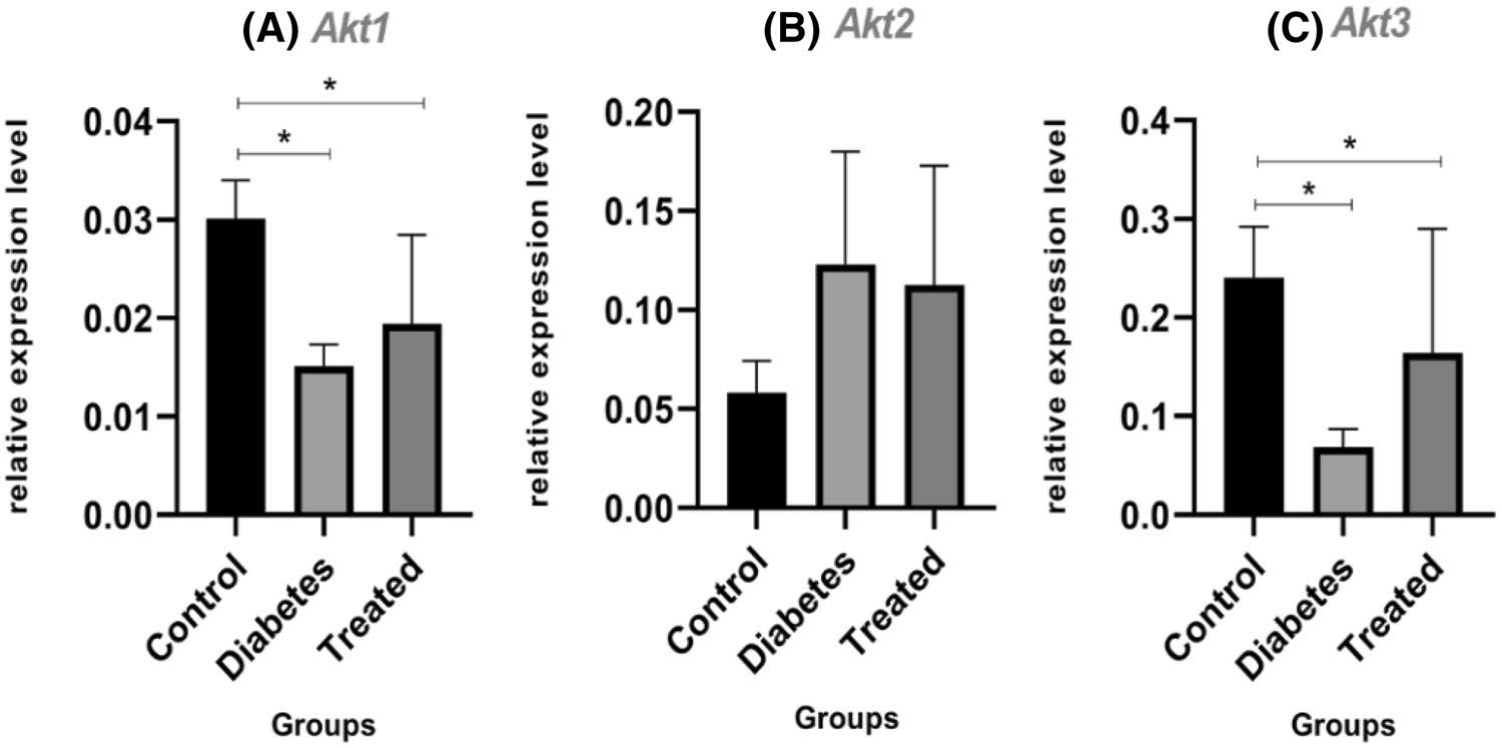
Relative expression of *Akt* genes in the hippocampus between control, diabetic and diabetic groups treated with RA, by real-time PCR. In the diabetic and treated groups, respectively, there was a significant down-regulation of 47% and 32% for *Akt1* compared to the control group. (*P < 0.05) (**A**). Comparison of the relative expression of *Akt3*, between the control group with the diabetic and treated groups showed a significant down-regulation of 67% and 20%, respectively (*P < 0.05) (**B**). There was, however, no significant difference in the *Akt2* gene expression between the three groups (*P* > 0.05) (**C**). The columns represent the mean ± SEM.

*Akt1* and *Akt3* expression in diabetic rats showed a significant downregulation of 47% (*P* = 0.0404) and 67% (*P* = 0.0407), respectively, when compared with the control group (Fig. 7A, C). Treating the diabetic rats with RA returned the expression of *Akt1* and *Akt3* relatively to the normal condition with *P* = 0.0231 and *P* = 0.0236, respectively (Fig. 7A, C). The expression of the *Akt2* was not significantly different among the three groups (Fig. 7B).

## Discussion

### Fructose as an important glycating agent

In hyperglycemia and diabetic conditions, glucose concentration increases in the insulin-independent tissues such as neural tissue, glomeruli, lens, and erythrocytes. Accordingly, the polyol pathway can be activated and sorbitol is converted to fructose by sorbitol dehydrogenase^6–8^. The high reactivity of fructose, either directly or through its metabolites, may contribute to the formation of intracellular glycation products and vascular complications^22^. Interestingly, fructose's contribution to the onset of these deleterious reactions is 5–8 times higher than that of glucose^23,24^. Briefly, first, fructose has more reactivity than other sugars. Second, in tissues where the sorbitol pathway is active, glucose is converted to fructose. Third, in diabetic conditions, the amount of fructose in the eye lens may increase by 23 times^25^, making glycation a more likely event in the body. Therefore, in the present study, fructose was used as a glycating agent for these reasons.

### DNA glycation and inhibition by rosmarinic acid

To determine the structural alteration of DNA during the reaction with fructose, UV spectroscopy and fluorescence study were employed. Ultraviolet spectroscopy is often used to determine the conformational changes of chromophores occurring in DNA^26–28^. 5, 10, and 15 days after glycation, the trend of the kinetic changes at 260 nm continued to increase. It could be concluded that fructose's modification of nitrogenous bases caused single-strand breakage, which resulted in the destruction of chromophore classes. As a result, the hypochromic phenomenon may be caused by dispersion force interactions between stacked chromophores, which could be explained in terms of extensive strand scission and intensity exchange between different transitions. Moreover, some slight red shifts at the peaks may result from the denaturation-dependent head-to-tail arrangement of the transition dipoles (Fig. 1). According to Table 1, which represents the difference in the ratio intensities of A260/A280 for the native and glycated DNA, it could be seen how fructation caused marked changes in the structure of the DNA generating light-absorbing molecules.

In the agarose gel electrophoresis study, alteration in the migration profile of fructose-DNA might originate from the significant damage in the amino groups of nucleotides at the advanced glycation stages (15 days incubation), causing further rearrangement and destabilizing the phosphodiester backbone. Modification of DNA by fructose gives rise to breaking single strands and generating nucleotide adducts, due to increased oxidative stress^21,29^. Moreover, the formation of superoxide radicals is involved in the Maillard reaction of DNA with fructose and other sugars^30^. The significant decrease in the single strand breakage for the RA treated samples could be attributed to its established antioxidant activity^19^. Reactive oxygen species (ROS) are a potent mediator causing cellular stress originating from sugars auto-oxidation^31,32^.

Generation of fluorogenic AGEs in the glycated-DNA samples was probed at the excitation/emission wavelength of 370/440 nm. Glycation of DNA by fructose generated fluorescent DNA_AGEs which could be characterized by the emission maxima of 440 nm. This is a typical event of DNA-AGEs that has been detected in vitro in the process of glycation^33^. This finding is also consistent with another study, suggesting that AGEs could be formed as the end-product of DNA molecules, resulting in the single-strand breakage^30^. The quenching effect of RA, as seen in Fig. 3 and Table 1, offers a clue for the reduction of advanced glycation end products during glycation. It suggested that the fructation of DNA resulted in the formation of nucleotide AGEs which could be associated with the increase in the mutation frequency and cytotoxicity^34^.

The results of the fluorescence microscope also confirmed those of the previous experiments. In the samples containing DNA-fructose, amorphous-like aggregates were observed (Fig. 4B); The presence of RA markedly diminished these insoluble aggregates (Fig. 4C) However, the reduction of the whole glycation-linked aggregation process by RA might originate from its preserving effect on the global fold of the DNA molecule. The occurrence of reactive Maillard intermediates with DNA may account for the strand scission and increased inter-strand crosslinking, culminating into adducts capable of forming yellow–brown fluorescent compounds^35,36^.

According to the zeta potential results, the particle sizes of the Fructose-DNA, RA–complex were comparable to that of the native DNA (Fig. 5). It is obvious that in the DNA-fructose system in which RA was present, the change in particle size caused by the addition of fructose was much smaller than that of the modified DNA (Fig. 5 and Table 1). Through these experiments, we found that the interaction of RA made the zeta-potential smaller than that in the fructose-DNA complex and the corresponding particle size obeyed the same trend.

From Table 1, the decrease in the Tm of the modified DNA could be ascribed to the generation of single-strand breaks and/or the altered hydrogen bonding between base pairs. The results were in the same trends of fluorescence intensity values (Table 1) obtained by AGE-specific fluorescence (λex 370 nm, λem 440 nm), thus largely representing the structure of the DNA change after adding fructose and protecting markedly by the applied RA.

Overall, our findings revealed that the fructation of DNA led to the formation of nucleotide adducts with increased oxidative stress. Thus, RA holds a considerable intervention potential with the underlying mechanism of fructose-mediated DNA-AGE formation. The inhibitory action of RA is partially due to its antioxidant potential and ROS scavenging activity; it could also be attributed to stacking with glycogenic nucleotides which direct the ligands to the glycogenic core, thereby counteracting the effect of the formed inter-strand cross-link in the duplex DNA. Since the mechanisms underlying the formation of advanced glycated-DNA may elevate the risk of cancer by enhancing the risk of mutagenesis, our results might help to design nutraceutical-based small molecules useful for decreasing the risk of mutation frequency and other DNA lesions leading to cytotoxicity.

### Diabetes, apoptosis in hippocampal neurons

The previous studies provide evidence that both types of diabetes are associated with functional and structural disorders in the brain^37–39^. One of the most critical disorders is apoptosis in hippocampal neurons^9–12^. Significant down-regulation expression of *Akt1* and *Akt3* (apoptosis inhibitor genes) in the current study also suggested the occurrence of apoptosis in the hippocampal neurons of STZ-induced diabetic rats.

### *Akt* genes with a distinct hippocampal expression

In the hippocampus, each *Akt* isoform has a distinct expression pattern: *Akt1* and *Akt3* are predominantly expressed in neurons, while *Akt2* is primarily expressed in astrocytes and glia^40^. *Akt2* deficiency is associated with insulin resistance, causing a diabetic syndrome with elevated plasma glucose levels; this suggests that *Akt2* is involved in the insulin signal^41^. These results, therefore, suggest that the reason for no significant difference in the mRNA level of *Akt2* in the present study could be the close relationship between the expression of this gene and insulin and blood sugar levels, as well as insulin signal. It is thought-provoking that astrocytes, unlike neurons, are insulin-dependent, and the present study emphasizes the possibility of DNA glycation in non-insulin-dependent cells.

### A link between apoptosis of hippocampal neurons in diabetic conditions and DNA fructation

Diabetes is associated with oxidative stress due to the increased free radical formation and the decreased activity of the antioxidant defense systems^42^. Hyperglycemia causes increased formation of Reactive Oxygen Species (ROS) through several pathways, including the polyol pathway and glycation^30,43^. Glycation and AGEs change the expression of genes^44,45^, as well as cause cell death^46^. According to these results, as well as those in previous sections, apoptosis in hippocampal neurons may be related to DNA damage induced by the polyol pathway's increased fructose concentration; In other words, apoptosis of the hippocampal neurons can be due to the glycation of DNA. Significant relative return of *Akt1* and *Akt3* expression to the normal state, based on the treatment of STZ-induced diabetic rats with RA as a natural phenol with the ability to inhibit glycation, confirms the possibility of apoptosis due to the DNA glycation.

## Conclusion

The findings of this research, confirm the anti-glycation properties of rosmarinic acid and point to it as a potential biophenol that can effectively minimize diabetes complications. According to the current study, hippocampal nerve cell apoptosis in diabetics may be due to DNA glycation. Therefore, further study into the likelihood of DNA glycation inside diabetic hippocampal neurons and other insulin-independent tissues is suggested to validate this phenomenon.

## Materials and methods

All of the chemicals used in this study, whose manufacturer is not listed in the text, are from Sigma Chemical Company, USA.

### Isolation of human DNA

Blood was drawn from healthy individuals. Blood was collected according to the instructions of the Ministry of Health of the Islamic Republic of Iran (Ethical number: IR.UI.REC.1399.056). Genomic DNA was isolated by optimization of the Proteinase K-Buffer method^47^, using lysis buffer A (11% sucrose, 1% Triton X-100, 5 mM MgCl2, 10 mM Tris pH 8) and buffer B (10 mM sodium citrate, 1% SDS, 10 mM Tris, 10 mM EDTA pH 8.0), from human blood.

### Preparation of glycated and treated DNA whit RA

In vitro glycation of DNA was done and characterized, as previously described^48^. To summarize, DNA (20 ng/µl final concentration) from the blood of non-diabetic individuals was incubated with 25 mM fructose at 37 °C for 5, 10, and 15 days in the presence or absence of RA (25 µM final concentration) in phosphate buffer saline (PBS). This fructose concentration correlates to the minimum concentration needed for in vitro DNA glycation^49^. It has also been shown that 25 µM rosmarinic acid reduces the glycosylation of human fibroblasts in vitro^50^. 0.02% (w/v) NaN3 was added to the solution and purified into a low protein-binding filter (-GV 0.22 µm filter unit, Millipore) to avoid bacterial contamination. Aliquots were taken from the DNA–fructose solution after each incubation period and extensively dialyzed against the sterile PBS at 4 °C to remove the free fructose molecules. Under the same conditions, pure DNA solution was used as the control sample.

### UV–visible spectroscopy

The UV–vis spectroscopy was used to confirm the structural changes induced in the human blood DNA. The ultraviolet absorption profile of the modified DNA (20 ng/µl final concentration), incubated with 25 mM fructose at 37 °C for 5, 10 and 15 days in the PBS, was recorded in the wavelength range of 200–400 nm on the UV-2100 spectrophotometer in a quartz cuvette with 1 cm path length^27^.

### Agarose gel electrophoresis

To evaluate the effect of glycation (fructation) on DNA, 5 µl of native, glycated, and treated DNA was mixed with 1 µl of the loading dye 6X buffer. The samples were loaded into the wells of 1.5% agarose gel and electrophoresed for 1.5 h at 100 v. The gels were stained with ethidium bromide (0.5 mg/ ml), viewed by illumination under UV light, and photographed.

### Fluorescence studies

Fluorescence measurements were made on a Cary-Eclipse spectrophotometer (Varian model, Australia). Sample mixtures showing absorbance changes were subjected to the excitation wavelength of 370 nm and the emission wavelengths were recorded at the range of 380–500 nm. Fluorescence spectra of the modified nucleotides and DNA were determined by the subtraction of the background fluorescence of fructose and its possible degradation product during prolonged incubation.

### Fluorescence microscopy

Fluorescence microscopic measurements were performed using ThT dye (λex 440 nm, λem 480 nm) to visualize the morphological structure of the inter-strand cross-links formed in the duplex DNA molecules and the inhibitory activity of biophenol^51^. ThT (32 μM) was mixed with native (at 25 °C) and glycated DNA samples in the absence and presence of biophenols. After 60 min incubation at room temperature, 15 μl of each sample was transferred on cleaned glass slides to be analyzed under fluorescence microscopy. The images were obtained with an Olympus IX71 fluorescence microscope equipped with a digital CCD camera at 20 and 40 objective magnifications.

### Zeta potential measurements

Zeta potential evaluation was done according to the method developed by Chetty and Singh^26^. To describe briefly, the zeta potential and average particle sizes of DNA, DNA/Fructose, and biophenol–DNA/Fructose were determined by using ZetasizerNano-ZS90 (Malvern Instruments, Ltd.UK). The sample solution of DNA/Fructose (1 mg/mL) and biphenol–DNA/Fructose was placed in the test vessel; the average of three repeats of the measurements was reported. The molar ratio of biophenol to DNA/Fructose in the mixed system was 1:1.

### Thermal denaturation (Tm measurement)

A temperature scan of 30–95 °C at an increment of 1 °C/min was performed with a Shimadzu UV-240 spectrophotometer fitted with a temperature programmer and controller assembly; The thermal denaturation of native, glycated, and RA DNA samples was evaluated under similar conditions. Absorbance change (260 nm) and the melting temperature (Tm) of the samples were reported^52^.

### Experimental animals and samples collection

Male rats (Rattus Norvegicus) with an average weight of 200–220 g were purchased from the Faculty of Pharmacy, University of Isfahan. In the animal room of the Faculty of Science, University of Isfahan., Iran, the animals were housed in a 12-h alternating light–dark cycle at a temperature of 21 ± 2 °C. All experiments were approved by the Ethics Committee of the University of Isfahan, Iran, (Ethical number: IR.UI.REC.1399.056); they were conducted according to the guide for the Internationally Accepted Principles for Animal Use and Care^53^. The rats were divided into three groups (n = 8): (1) Control group, (2) diabetic group, and (3) diabetic group treated with RA. Sixteen animals were fasted for 24 h, and diabetes was induced using a single intraperitoneal injection (i.p.) of Streptozotocin (STZ) (45 mg/kg, freshly dissolved in 0.1 M citrate buffer, pH 4). Three days later, fasting blood glucose levels were determined using tail blood. Only rats were considered diabetic if basal blood glucose levels exceeded 250 mg/dl. After confirmation of diabetes, eight rats in the diabetic group received 30 mg/kg RA (mixed in deionized water), once daily by oral gavage for 8 weeks. This concentration of RA shows anti-oxidant and anti-glycate effects in diabetic rats by reducing the formation of MDA and advanced glycation end products^54^. Rats in the control groups were given an equal volume of water. The animals were anesthetized with a mixture of pentobarbital sodium and phenytoin sodium (Euthanasia III) at the end of week 8. Rats were sacrificed when they failed to respond to a toe pinch. The hippocampus tissue samples were isolated and flash frozen in liquid nitrogen; they were preserved at − 80 °C until further experiments.

### Total RNA extraction and reverse transcription

Total RNA was extracted from the hippocampus tissue samples using Trizol reagent, according to the manufacturer's instructions. The extracted RNA was dissolved in DEPC-treated water. Gel electrophoresis was used to check the quality of the isolated RNA; also, Nanodrop (Thermo fisher-Onec) was used to determine the RNA concentration. Reverse transcription was conducted on 1000 nanograms of the total RNA in a final volume of 20 µl. Under the conditions suggested by the supplier (Thermo Fisher), cDNA was synthesized using the random hexamer primers and MMLV-reverse transcriptase.

### Primer design for real-time PCR

The primers were designed using Oligo 7 and Beacon Designer 8 software. For *Akt1* (NM_033230.2), *Akt2* (NM_017093.1), *Akt3* (NM_031575.1), and Beta-actin (NM_031144.3), the specific primers should result in a 156, 127, 107, and 123 bps product, respectively. The primer sequences are listed in Table 2.

**Table 2:** Primer used for the real-time PCR.

Gene	Primer for real-time PCR
*Akt1* (NM_033230)	5′-TCCCTTCCTTACAGCCCTCAAG-3′—sense 5′-GACACAATCTCCGCACCGTAG-3′—antisense
*Akt2* (NM_017093)	5′-TTCCTTACAGCCCTGAAGTATGCC-3′—sense 5′-GTGCCCGATCCTCCGTGA-3′—antisense
*Akt3* (NM_031575)	5′-GAACGACCAAAGCCAAATACA-3′ —sense 5′-TCTGTCCATTCTTCTCTTTCCT-3′—antisense
*Beta-actin* (NM_031144)	5′-CTCTATGCCAACACAGTG-3′—sense 5′-AGGAGGAGCAATGATCTT-3′—antisense

### *Akt* genes expression analysis by real-time PCR

For the amplification of *Akt*, 1 µl cDNA was added to the SYBRGreen Master Mix (Ampliqon) containing the specific primers. Real-time PCR was performed in a Bio-Rad thermocycler. The same total volume (12 µl) and thermal settings were used for all genes: 5 min of pre-incubation at 95 °C and this was followed by 40 cycles of 30 s at 95 °C, 30 s at 58 °C, and 30 s at 72 °C. The melting curve plot was drawn between 55 and 95 °C. Triplicates of each sample were run.

### Statistical analysis

All data were presented as means ± standard deviation (SD). Statistical analysis was performed by one-way ANOVA, using GraphPad Prism software, version 7. A Duncan's post-hoc comparison was then made to analyze the sources of significant differences by SAS, version 9.2. A *P*-value ˂ 0.05 was considered statistically significant.

### Ethical approval

The study was approved by the Ethics Committee of Research, University of Isfahan, Iran (IR.UI.REC.1399.056). Collecting blood samples was performed in accordance with the seventh edition of the Helsinki declaration and its later amendments or comparable ethical standards and the research protocol and written consent was first submitted to and confirmed by the Ethics Committee of Research, University of Isfahan. Written informed consent was collected from each blood donor and the full explanation of the study, including the aims, methods, and sources of funding, institutional affiliations of the researchers, the anticipated benefits, and post-study provisions, was provided to each blood donor. It should be noted that the donors had no diabetes and were over 18 years of age.

Also, the present study was approved by the Institutional Animal Care and Use Ethics Committee of the University of Isfahan, Iran, (Ethical number: IR.UI.REC.1399.056) and carried out in compliance with the ARRIVE guidelines. The method of caring for mice as well as their anesthesia is described in the materials and methods section, experimental animals and, samples collection.
